# Supplementary figures and images for: Integrating machine learning with otolith isoscapes: Reconstructing connectivity of a marine fish over four decades
Source: PLoS One. 2023 May 31;18(5):e0285702. doi: 10.1371/journal.pone.0285702 (PMC10231828; doi:10.1371/journal.pone.0285702)

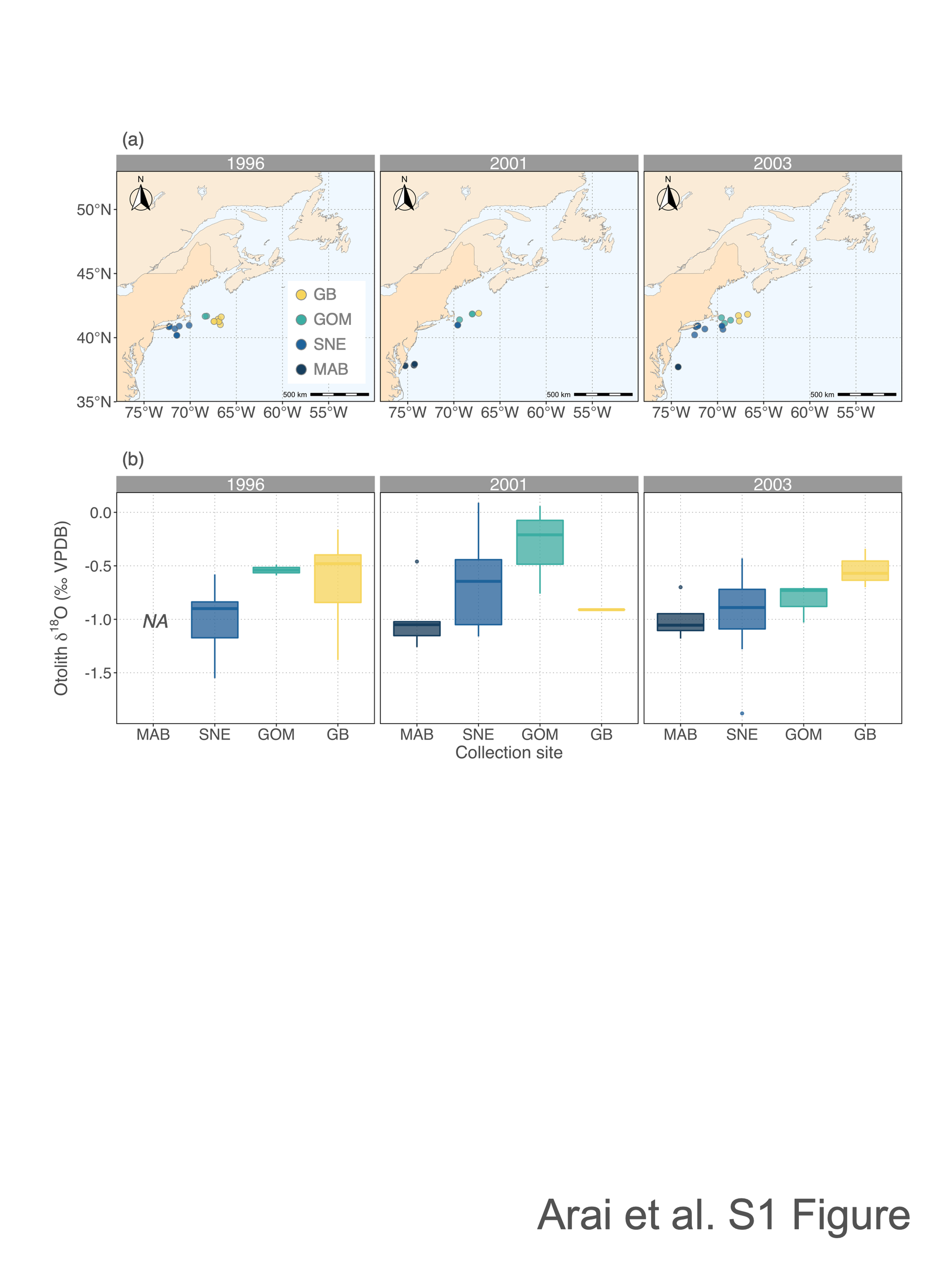

Supplement: S1 Fig — Collection sites (a) and measured otolith oxygen stable isotope values (b) of age-0 juvenile Northwest Atlantic mackerel collected in 1996, 1999, and 2003. GB = Georges Bank, GOM = Gulf of Maine, SNE = Southern New England, MAB = Mid-Atlantic Bight. The map was created using the rworldmap package [64] in R. (TIF) [file pone.0285702.s001.tif]

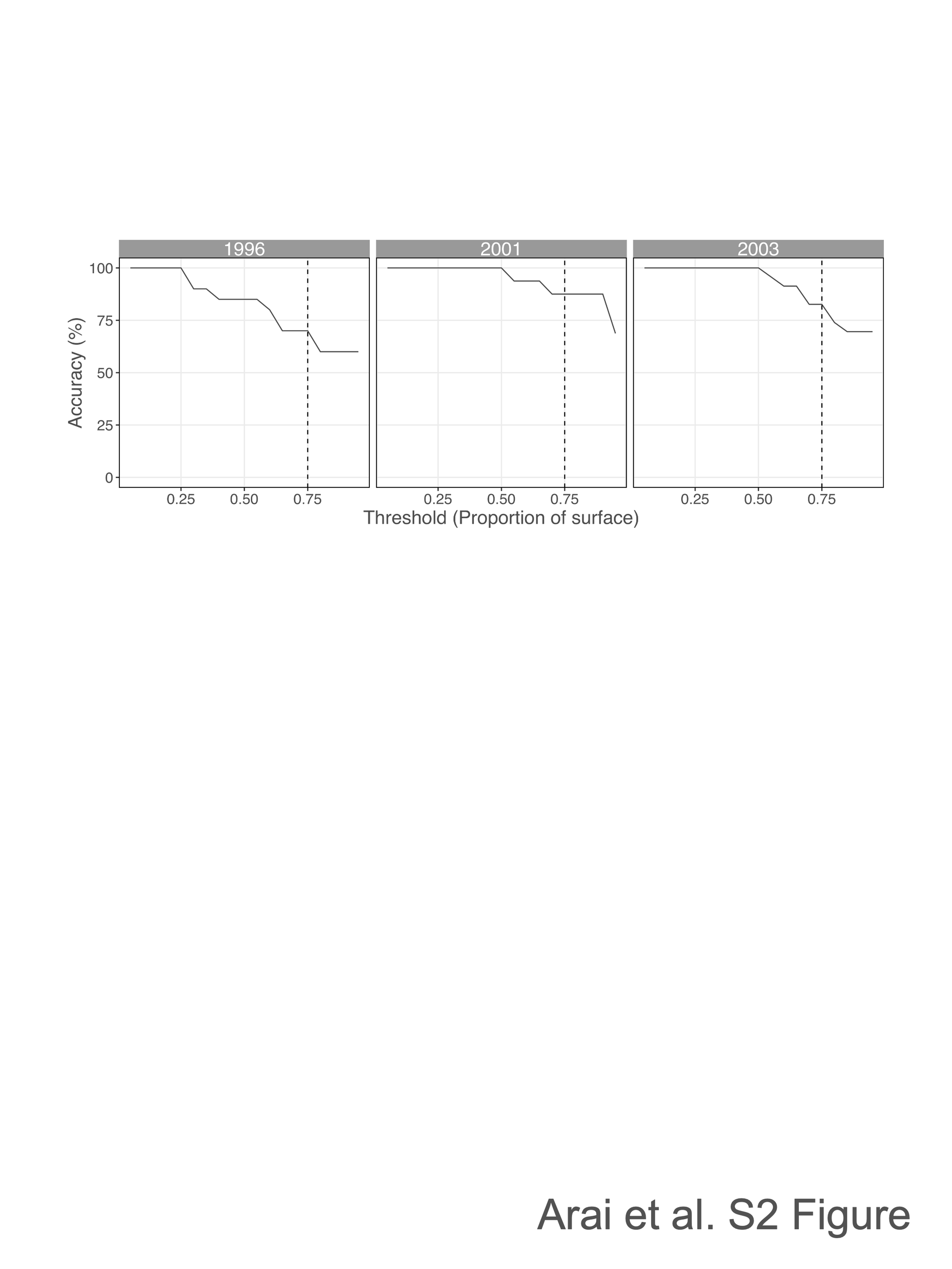

Supplement: S2 Fig — Accuracy was computed over a range of thresholds (i.e., precision) from 0.05 to 0.95. The 75th percentile threshold (dotted vertical line) provided a balanced accuracy-precision trade-off and was selected for binary transformation, where grid cells with posterior probabilities in the upper 25% of all grid cells were assigned “likely” and the remaining 75% as “unlikely” location of origin. (TIF) [file pone.0285702.s002.tif]
