# Supplementary material for: Integrating machine learning with otolith isoscapes: Reconstructing connectivity of a marine fish over four decades
Source: PLoS One. 2023 May 31;18(5):e0285702. doi: 10.1371/journal.pone.0285702 (PMC10231828; doi:10.1371/journal.pone.0285702)
Supplement: S1 Table — Data for year-classes 1998–2000 and 2011 are from Redding et al. [61], and data for year-classes 2012–2016 are from Arai et al. [63]. (DOCX) [file pone.0285702.s003.docx]

**S1 Table.** **Northwest Atlantic mackerel otolith sample size by year-class, sampling period, collection site, and data type. Data for year-classes 1998–2000 and 2011 are from Redding et al. [61], and data for year-classes 2012–2016 are from Arai et al. [63].**

| **Year-class** | **Years collected** | **Collection site** | **Sample size** |  |  |
| --- | --- | --- | --- | --- | --- |
|  |  |  | **Northern baseline** | **Southern baseline** | **Unknown adult** |
| 1973 (N = 61) | 1974, 1975 | Canada | 20 |  |  |
|  |  | Gulf of Maine |  |  |  |
|  |  | Georges Bank |  | 20 | 6 |
|  |  | Southern New England |  |  | 15 |
|  |  | Mid-Atlantic Bight |  |  |  |
|  |  | US (subregion unknown) |  |  |  |
| 1974 (N = 74) | 1975, 1976, 1978 | Canada | 20 |  |  |
|  |  | Gulf of Maine |  |  |  |
|  |  | Georges Bank |  |  | 20 |
|  |  | Southern New England |  | 20 |  |
|  |  | Mid-Atlantic Bight |  |  | 14 |
|  |  | US (subregion unknown) |  |  |  |
| 1998 (N = 102) | 1999–2001, 2003 | Canada | 28 |  |  |
|  |  | Gulf of Maine |  |  |  |
|  |  | Georges Bank |  | 18 | 17 |
|  |  | Southern New England |  | 14 | 20 |
|  |  | Mid-Atlantic Bight |  |  | 5 |
|  |  | US (subregion unknown) |  |  |  |
| 1999 (N = 107) | 2000, 2001, 2003 | Canada | 27 |  |  |
|  |  | Gulf of Maine |  |  |  |
|  |  | Georges Bank |  | 19 | 18 |
|  |  | Southern New England |  | 8 | 32 |
|  |  | Mid-Atlantic Bight |  |  | 3 |
|  |  | US (subregion unknown) |  |  |  |
| 2000 (N = 66) | 2001, 2003 | Canada | 12 |  |  |
|  |  | Gulf of Maine |  |  |  |
|  |  | Georges Bank |  | 24 | 2 |
|  |  | Southern New England |  | 15 | 7 |
|  |  | Mid-Atlantic Bight |  |  | 6 |
|  |  | US (subregion unknown) |  |  |  |
| 2011 (N = 89) | 2012–2015 | Canada | 9 |  |  |
|  |  | Gulf of Maine |  | 6 | 23 |
|  |  | Georges Bank |  | 10 | 14 |
|  |  | Southern New England |  | 4 | 6 |
|  |  | Mid-Atlantic Bight |  |  | 17 |
|  |  | US (subregion unknown) |  |  |  |
| 2012 (N = 100) | 2013–2016 | Canada | 40 |  |  |
|  |  | Gulf of Maine |  | 9 | 7 |
|  |  | Georges Bank |  | 2 | 18 |
|  |  | Southern New England |  |  | 2 |
|  |  | Mid-Atlantic Bight |  |  | 18 |
|  |  | US (subregion unknown) |  | 4 |  |
| 2013 (N = 99) | 2014–2017 | Canada | 40 |  |  |
|  |  | Gulf of Maine |  | 3 |  |
|  |  | Georges Bank |  |  |  |
|  |  | Southern New England |  | 11 |  |
|  |  | Mid-Atlantic Bight |  |  | 45 |
|  |  | US (subregion unknown) |  |  |  |
| 2014 (N = 100) | 2015–2018 | Canada | 40 |  |  |
|  |  | Gulf of Maine |  | 15 |  |
|  |  | Georges Bank |  |  |  |
|  |  | Southern New England |  |  | 12 |
|  |  | Mid-Atlantic Bight |  |  | 33 |
|  |  | US (subregion unknown) |  |  |  |
| 2015 (N = 112) | 2015–2019 | Canada | 40 |  |  |
|  |  | Gulf of Maine |  | 11 | 12 |
|  |  | Georges Bank |  |  | 2 |
|  |  | Southern New England |  | 4 | 28 |
|  |  | Mid-Atlantic Bight |  |  | 11 |
|  |  | US (subregion unknown) |  |  | 4 |
| 2016 (N = 52) | 2017–2019 | Canada | 19 |  |  |
|  |  | Gulf of Maine |  | 2 | 1 |
|  |  | Georges Bank |  | 8 | 2 |
|  |  | Southern New England |  | 8 | 4 |
|  |  | Mid-Atlantic Bight |  |  | 1 |
|  |  | US (subregion unknown) |  |  | 7 |
