# Supplementary material for: Integrating machine learning with otolith isoscapes: Reconstructing connectivity of a marine fish over four decades
Source: PLoS One. 2023 May 31;18(5):e0285702. doi: 10.1371/journal.pone.0285702 (PMC10231828; doi:10.1371/journal.pone.0285702)
Supplement: S2 Table — (DOCX) [file pone.0285702.s004.docx]

**S2 Table.** **Hyperparameters selected for six machine learning classifiers using the grid search method on 10 cross-validation folds.**

| **Classifier** | **Hyperparameters** | |
| --- | --- | --- |
| Artificial Neural Networks (ANN) | Number of Hidden Units: 10 | Amount of Regularization: 0.00001 |
| Decision Trees (DT) | Cost-Complexity: 0.00001 | Tree Depth: 13 |
| *k*-Nearest Neighbors (*k*NN) | Number of Neighbors: 3 | – |
| Naïve Bayes (NB) | Kernel Smoothness: 0.8 | Laplace Correction: 1.3 |
| Random Forest (RF) | Number of Trees: 3000 | Minimal Node Size: 3 |
| Support Vector Machines (SVM) | Cost: 3 | – |
