# Supplementary material for: Integrating machine learning with otolith isoscapes: Reconstructing connectivity of a marine fish over four decades
Source: PLoS One. 2023 May 31;18(5):e0285702. doi: 10.1371/journal.pone.0285702 (PMC10231828; doi:10.1371/journal.pone.0285702)
Supplement: S3 Table — (DOCX) [file pone.0285702.s005.docx]

**S3 Table. Age-0 juvenile Northwest Atlantic mackerel otolith sample size by year-class, sampling period, and collection site.**

| **Year-class** | **Years collected** | **Collection site** | **Sample size** |
| --- | --- | --- | --- |
| 1996 (N = 20) | 1996 | Gulf of Maine | 2 |
|  |  | Georges Bank | 8 |
|  |  | Southern New England | 10 |
|  |  | Mid-Atlantic Bight |  |
| 2001 (N = 16) | 2001 | Gulf of Maine | 3 |
|  |  | Georges Bank | 1 |
|  |  | Southern New England | 6 |
|  |  | Mid-Atlantic Bight | 6 |
| 2003 (N = 23) | 2003 | Gulf of Maine | 3 |
|  |  | Georges Bank | 3 |
|  |  | Southern New England | 13 |
|  |  | Mid-Atlantic Bight | 4 |
