# Supplementary material for: Integrating machine learning with otolith isoscapes: Reconstructing connectivity of a marine fish over four decades
Source: PLoS One. 2023 May 31;18(5):e0285702. doi: 10.1371/journal.pone.0285702 (PMC10231828; doi:10.1371/journal.pone.0285702)
Supplement: Attachment — Submitted filename: Reviewer comments.docx [file pone.0285702.s006.docx]

This study proposed and described a novel framework to delineate the geographic origin and nursery hotspots over a spatiotemporal scale for the Northwest Atlantic mackerel by means of otolith stable isotope concentrations, which could be a suitable and interesting approach for other marine species as well.

This paper presents the results of **original research** with an important contribution to the field of stock management informed otolith microchemistry presented as a high-quality manuscript which significant importance for the broad readership of *PLOS ONE* journal. The manuscript contains all the relevant information on the field, and is easy to follow, the motivation behind the work is clearly described, with experiments, statistics, and other analyses are performed to a **high technical standard and are described in sufficient detail,** with straight and sound **conclusions that are supported by the data**. The manuscript is easy to follow and is presented in **presented in an intelligible fashion and is written in standard English**. Authors do state that **meet all applicable standards for the ethics of experimentation and research integrity**, and that **all data are fully available without restriction**.

However, there are some minor issues that could be improved in my point of view before its publication as a high impact article. I have provided general comments and suggestions for each section of the manuscript, followed by specific comments to the authors that should be quite straightforward to address, I hope authors find them useful. If authors can address these minor issues, I do believe that this manuscript will be of great value as a publication for *PLOS ONE* journal. Finally, my most sincere congratulations for this nice piece of work, this is good science that needs to be shared!

**INTRODUCTION**

The introduction provides the reader an adequate background and clearly described the motivation behind this work. However, I do fill that too much weight is given (lines 86-99) on how the analysis has been done, which is perfectly described in M&M sections, and I do miss few more background on otolith stable isotopes composition, e.g. how oxygen composition of otolith aragonite responds to variations in the environment and why it is useful. If you could balance the text between both topics it would be very nice.

*Specific comments to the authors:*

*Line 53: I would suggest replacing “internal tags” for something like “natural markers” as internal tags usually refers to fish tagging devices and can be confusing.*

*Line 54: I would suggest starting the second sentence with an “Here” or sth similar to emphasize that you are still talking about chemical markers in biogenic tissues, as if not, it could be again misunderstood as the most common approach for tracking highly migratory fish is by archival or pop up tagging. Something like “Here, the most common approach consists of reconstructing…”*

*Line 63: You can cite Jones et al. 2017 here to reinforce this statement.*

*Jones, C. M., Palmer, M., & Schaffler, J. J. (2017). Beyond Z ar: the use and abuse of classification statistics for otolith chemistry. Journal of Fish Biology, 90(2), 492-504.*

*Lines 63-66: This is true because most older papers used traditional approaches, but I would also note that there is a trend towards change e.g.*

*Wright, P. J., Régnier, T., Gibb, F. M., Augley, J., & Devalla, S. (2018). Assessing the role of ontogenetic movement in maintaining population structure in fish using otolith microchemistry. Ecology and Evolution, 8(16), 7907-7920.*

*Zhang, C., Ye, Z., Li, Z., Wan, R., Ren, Y., and Dou, S. (2016). Population structure ofJapanese Spanish mackerel Scomberomorus niphonius in the Bohai Sea, the Yellow Sea and the East China Sea: evidence from random forests based on otolith features. Fisheries Science 82, 251–256. doi:10.1007/S12562-016-0968-X*

*Artetxe-Arrate, I., Fraile, I., Crook, D. A., Zudaire, I., Arrizabalaga, H., Greig, A., & Murua, H. (2019). Otolith microchemistry: a useful tool for investigating stock structure of yellowfin tuna (Thunnus albacares) in the Indian Ocean. Marine and Freshwater Research, 70(12), 1708-1721.*

*Lines 72-73: Not sure if completely agree, don’t always explicitly incorporate all sources of variation into account. E.g. individual physiology for stable isotope incorporation*

*Line 80: Perhaps “Combined” instead of “Hybrid”?*

*Line 82: I think that is not correct to use the term “highly migratory marine species here”. Highly migratory species are legally defined as those listed in Annex 1 of UNCLOS (https://www.un.org/depts/los/convention_agreements/texts/unclos/annex1.htm) and Atlantic mackerel is not part of this list. Please adapt this sentence here and elsewhere in the manuscript.*

**MATERIAL AND METHODS**

The section provides enough detail on the technique, equations and statistical approaches used so that other researchers can reproduce them. I just miss a little bit more information regarding the Study species, such as how it is managed (e.g. Atlantic mackerel managed separately in US and Canada?) and spawning conditions that feel important to known, such as when are they capable of spawning, age or length, so that then it is better understood why you separate age-1 as baseline and age>2 for assignment purposes. I also would recommend to include a summary Table with the individuals that have been analysed, giving information numbers in the, 201 baseline, which are for assignment, how much fish belong to each year, how much fish were collected from each fishery etc and also present the age-0 juvenile samples used for testing the accuracy of the isoscape.

*Specific comments to the authors:*

*Line 102: See comment above about “highly migratory”, perhaps you can find another way to explain this, or go a bit further and explain here the migrations that Atlantic mackerel undergoes (e.g. from spawning grounds in XX towards feeding grounds in XX).*

*Line 109: What does “dominant” imply here? Higher production, biomass…?*

*Line 118: I would suggest removing “(or ear stone)” from here, and if you prefer, to move this to the Introduction.*

*Lines 137 and 139: I cannot see what you are referring to in Fig.1. Perhaps a table could help.*

*Line 142: Why do you say that age-1 are known origin? Please can you add more information that support this statement, e.g. they do not migrate until age X, or they have shown to have limited movements outside nursery areas….*

*Lines 158-159: Can you explain more on the implications of including some age-2 northern contingents in the baseline? Where they used again for assignments or not? Does Atlantic mackerel show some spawning site fidelity?*

*Lines 161: Was Multivariate normality, linearity and multicolinearity and homoscedasticity of otolith 18O and 13C data tested prior MANOVA analyses?*

*Line 245: If possible, could you please add which depth range are you considering here as “subsurface” (e.g 0-5 m depth)..*

**RESULTS**

Overall results are well presented and support the data, and are reported in a concise, straightforward manner, using tables or figures when appropriate. Figure captions are very well described so that they are easy to interpret.

*Specific comments to the authors:*

*Line 341-343: Are there significant differences between contingents in every year? I think it will be nice an interesting to analyse this option too, as there are years (e.g. 1973, 1974, 2011, 2016…) were there it seems not to be differentiation, and this is also important for the discussion.*

*Line 343-344: Less distinction or no distinction?*

*Line 347: From the Figure 3 you cannot appreciate the fact that northern contingents from 1973 1974, 2011,2013 and 2014 for instance had lower oxygen values.*

*Lines 357: What is the threshold for low moderate and high? Not sure if needed but think it will improve readers experience.*

*Line 395-396: From figure 5 it seems that northern contingents were not prevalent in 2011, 2012 and 2016, please clarify this.*

*Lines 400-403: This paragraph is a little bit confusing for me, because I do not have very clear were this numbers come from.*

*Figure 6: I think it could facilitate to the reader if you can add the locations you mentioned in the text in lines 415-425.* ***This is just a suggestion****, but as you have 4 x3 plots and a blank space, you can use this black space to draw the same map without predictions but with location names, so then it is easier to follow the rest of the maps. If not, one can always return to Figure 1.*

**DISCUSSION**

Authors place into context observed results and highlight the most important and discuss the implications of this study into a broader context, which is very interesting and nice. It provides a good overview and closure of the manuscript, with conclusions aligning with the aim of the proposed research. However, I do miss some more further discussion on the potential limitations of the approach and some mention to he Suess effect into otolith C13 data.

*Specific comments to the authors:*

*Line 495: I suggest replacing “hybrid” by “combined”*

*Line 529: Perhaps “primarily driven by changes in temperature”*

*Line 559: I suggest replacing “tissue” here by “biogenic”, “hard-calcareous structures” or others here.*

*Line 562: But see Martino et al. 2022*

*Martino JC, Trueman CN, Mazumder D, Crawford J, Doubleday ZA. The universal imprint*

*of oxygen isotopes can track the origins of seafood. Fish Fish. 2022. doi:10.1111/faf.12703*
